# Supplementary figures and images for: Protective Effect of Alpha-Lipoic Acid on Salivary Dysfunction in a Mouse Model of Radioiodine Therapy-Induced Sialoadenitis
Source: Int J Mol Sci. 2020 Jun 10;21(11):4136. doi: 10.3390/ijms21114136 (PMC7312690; doi:10.3390/ijms21114136)

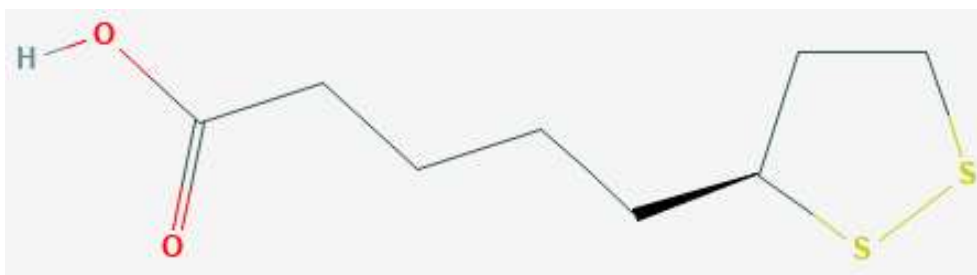

**Supplementary Figure S1.** Chemical structure of alpha lipoic acid.

Supplement: Supplementary file 1 [file ijms-21-04136-s001.pdf]
